# Supplementary material for: Defect-mediated ripening of core-shell nanostructures
Source: Nat Commun. 2022 Apr 25;13:2211. doi: 10.1038/s41467-022-29847-8 (PMC9038757; doi:10.1038/s41467-022-29847-8)
Supplement: Supplementary file 1 — Supplementary Information [file 41467_2022_29847_MOESM1_ESM.pdf]

Supplementary Information

Defect-mediated ripening of core-shell nanostructures

Qiubo Zhang[1#], Xinxing Peng[2#], Yifan Nie[1], Qi Zheng[1], Junyi Shangguan[1, 3], Chao Zhu[4], Karen C. Bustillo[2], Peter Ercius[2], Linwang Wang[1], David T. Limmer[1,5,6,7], and Haimei Zheng[1, 3 *]

[1] Materials Science Division, Lawrence Berkeley National Laboratory, Berkeley, CA 94720, USA

[2] National Center for Electron Microscopy, Molecular Foundry, Lawrence Berkeley National Laboratory, Berkeley, CA 94720, USA

[3] Department of Materials Science and Engineering, University of California, Berkeley, CA 94720, USA

[4] School of Materials Science and Engineering, Nanyang Technological University, Singapore, 639798, Singapore

[5] Department of Chemistry, University of California, Berkeley, CA 94720, USA

[6] Chemical Science Division, Lawrence Berkeley National Laboratory, Berkeley, CA 94720, USA

[7] Kavli Energy Nanoscience Institute, Berkeley, CA 94720, USA

[#] These authors contributed equally to this work

[*]Correspondence to: hmzheng@lbl.gov (H.Z.)

This Supplementary Information file includes:
Supplementary Figure 1 to Supplementary Figure 14
Supplementary Tables 1 to Supplementary Tables 2

Other Supplementary Materials include the following:
Supplementary Movies 1 to Supplementary Movies 6

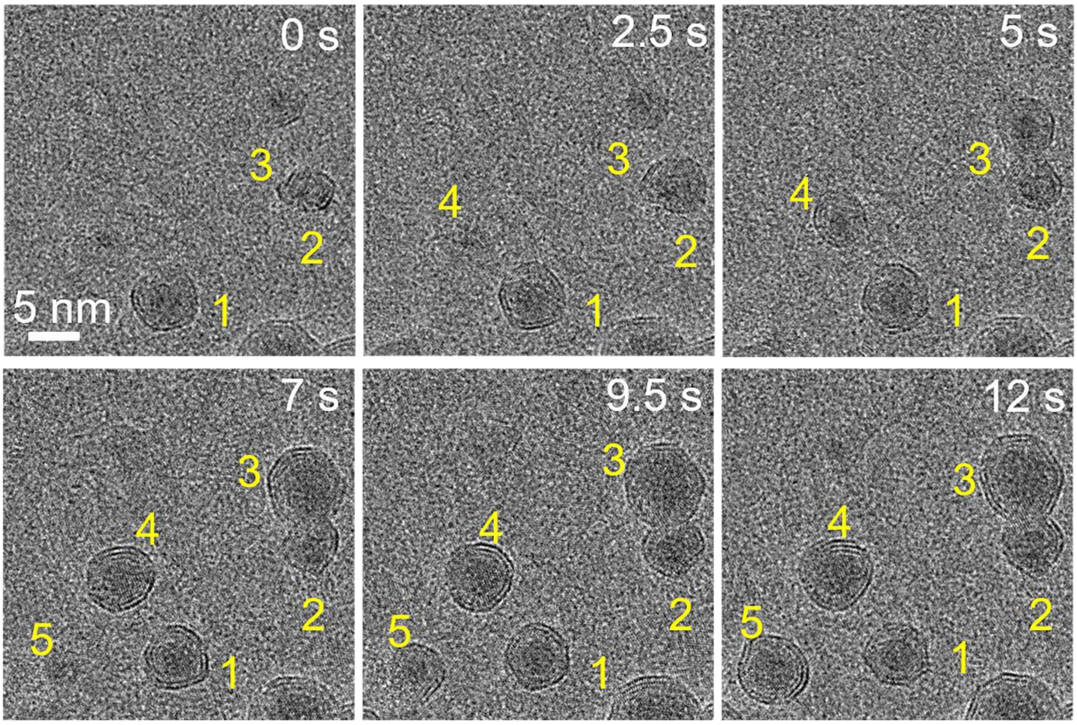

Supplementary Fig. 1: In-situ formation of Cd-$CdCl_2$ core-shell structured particles in the growth solution. The Cd-$CdCl_2$ core-shell is formed in situ by irradiating the growth solution with an electron beam. At the initial stage, there are only three core-shell particles in the image. Over time, the original small particles become larger, and new particles are produced in the solution. $CdCl_2$ precipitates out of the growth solution and adsorbs on the surface of the Cd core spontaneously.

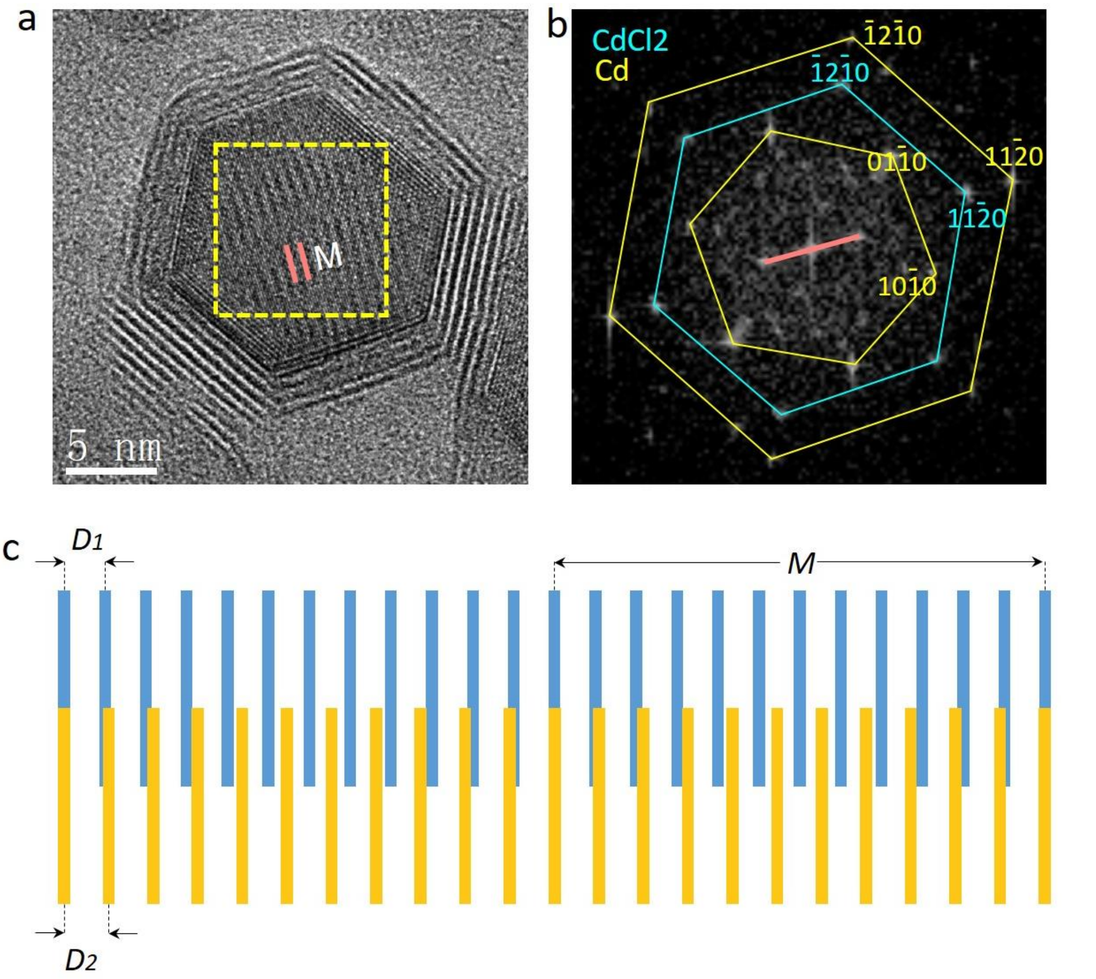

Supplementary Fig. 2: Determination of a $CdCl_2$ shell covered the (0001) facet of the core. a, A HRTEM image of the core-shell structured particle (Particle 1). We can clearly see the difference between the core and shell lattice fringes to determine the core-shell structure from the image. Some moiré fringes are visible in the core portion, indicating that there should be a shell covering the base surface of the Cd-$CdCl_2$ core-shell structured particle. **b,** The corresponding FFT pattern of the yellow square area in **(a)**. The yellow-marked diffraction spots belong to the hexagonal cadmium structure, and the blue-marked diffraction spots belong to the trigonal cadmium chloride structure. The $\langle 11\bar{2}0 \rangle$ direction of the Cd is parallel to the $\langle 11\bar{2}0 \rangle$ direction of the $CdCl_2$. Two diffraction points marked in red correspond to the Moiré pattern, the spacing is 0.66 nm. **c,** Moiré pattern formation diagram shows that the Moiré pattern of 0.66 nm space is caused by the $\langle 11\bar{2}0 \rangle$ lattice fringes of Cd and the $\langle 11\bar{2}0 \rangle$ lattice fringes of $CdCl_2$. The interspace of Cd ($11\bar{2}0$) facets is D_1= 0.14885 nm, the interspace of $CdCl_2$ ($11\bar{2}0$) facets is D_2= 0.192295 nm. The calculated value of the Moiré pattern spacing (M_c=0.659 nm) is in good agreement with the observation (M=0.66). Therefore, there is a $CdCl_2$ shell lies on the (0001) facet of the core.

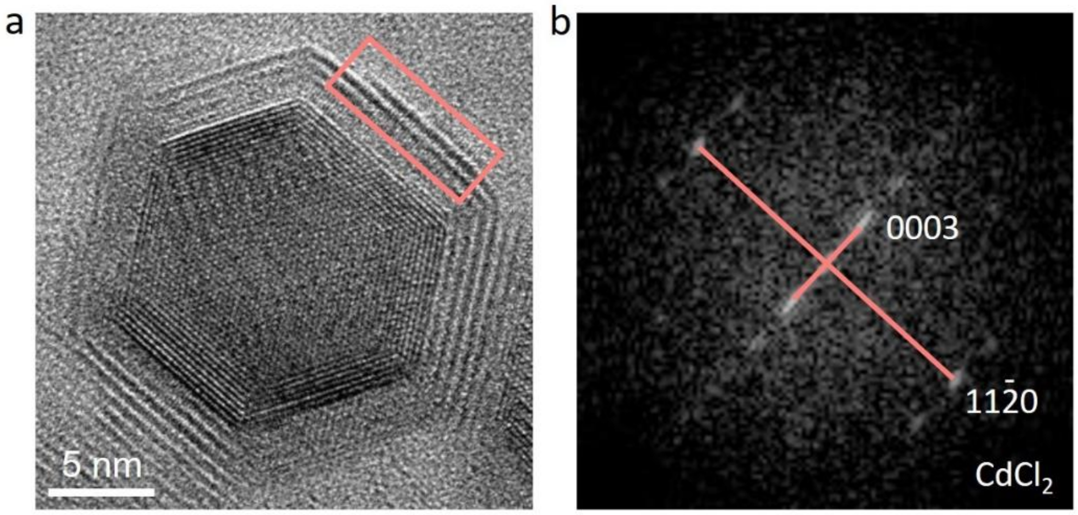

Supplementary Fig. 3: Structural characterization of the shell. a, A HRTEM image of the core-shell particle (Particle 1) viewed along the [$10\bar{1}0$] direction of Cd. **b,** FFT pattern corresponding to the pink square area in a. The FFT spots correspond to (0003) lattice spacing and (1120) lattice spacing of $CdCl_2$. The incident direction is along [$\bar{1}\bar{1}20$].

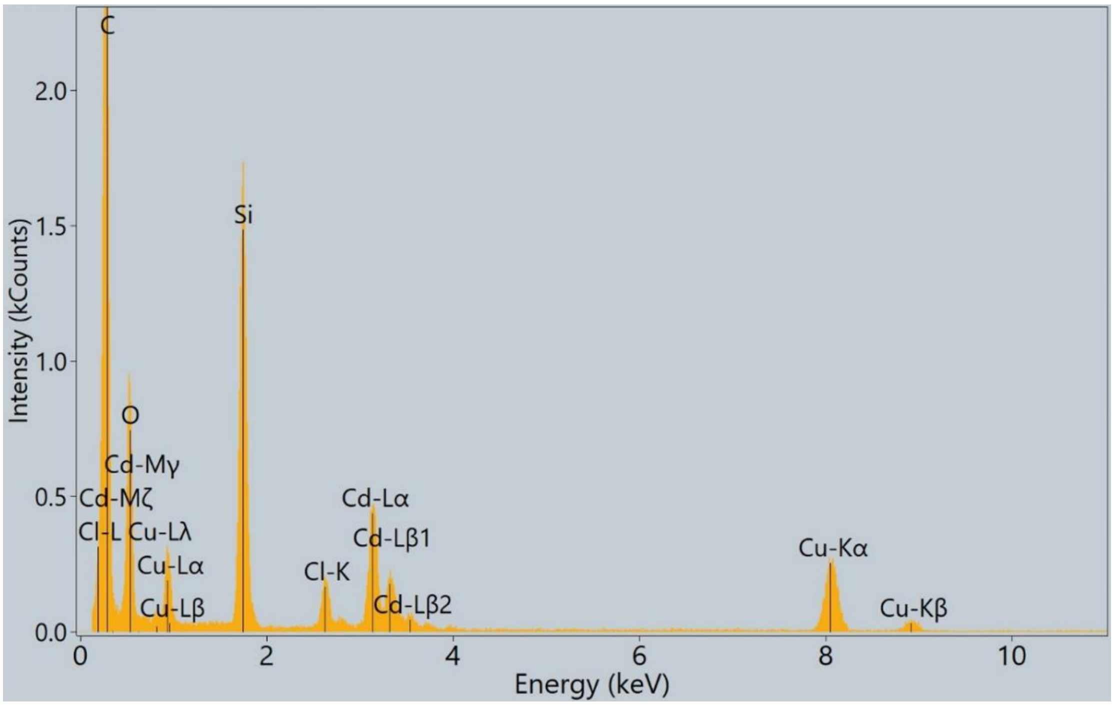

Supplementary Fig. 4: EDX spectrum of Cd-$CdCl_2$ core-shell particles. The EDX spectrum of Cd-$CdCl_2$ core-shell particles in Fig. 1i, which shows the presence of cadmium and chloride in the particles. The Cu peak comes from the Cu grid, and the Si peak comes from the tip of the TEM holder.

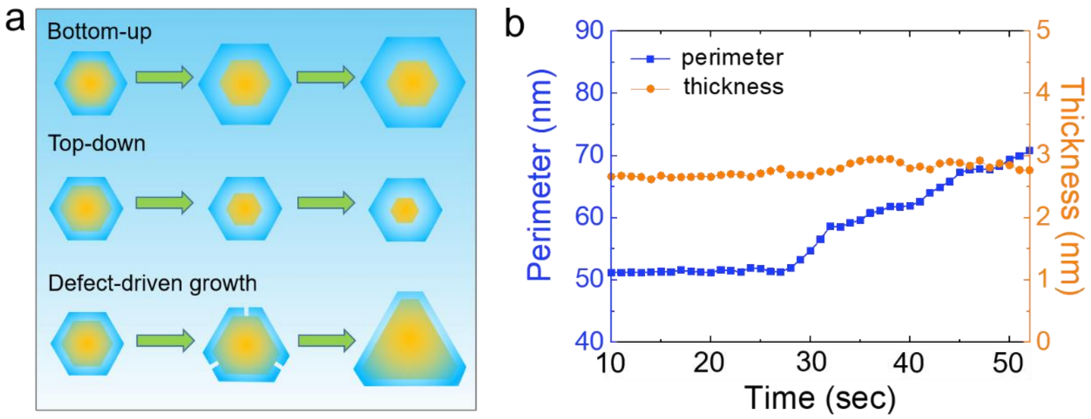

Supplementary Fig. 5: The growth of particle 1 follows a nontraditional path during the ripening. a, Schematic diagrams of defect-mediated growth, bottom-up growth, and top-down growth of the core-shell structured particles. The classic growth approaches of the CSP have two types: bottom-up and top-down. In the bottom-up growth methods, a core is usually used as a precursor material, and a shell is formed on the surface of the core and then grows radially outward. During the growth, the core size remains, and the shell continues to thicken. For the top-down method, a shell is formed beneath the core surface by oxidation or ion exchange reaction, and then it grows inward. In this case, the outward layer increases meanwhile consuming the core, resulting in a thicker shell and a smaller core. However, for the defect-driven growth, the growth of the CSP follows an entirely different pathway: the core keeps growing through cracking defects, and the perimeter of the shell increases correspondingly, but the thickness is almost unchanged. In this way, we can directly grow the core while maintaining the thickness of the shell to control the shape of the core-shell structure. **b,** Change of the perimeter and thickness of shell 1 during the defect-dominated growth process. We first measured the perimeter of the core (P_c) and the particle (P_p) in the projected image, then calculated the mean value ($P_s = \frac{1}{2}(P_c + P_p)$) as the perimeter of the shell. The thickness of the shell (T_S) is equal to the area of the particle (S_P) minus the area of the core (S_C) in the projected image, then divided by the perimeter of the shell (P_S), the equation is： $T_S = \frac{S_P - S_C}{P_S}$. Only the data between 10 and 50 seconds is collected because particle 1 has barely changed in the first 10 seconds.

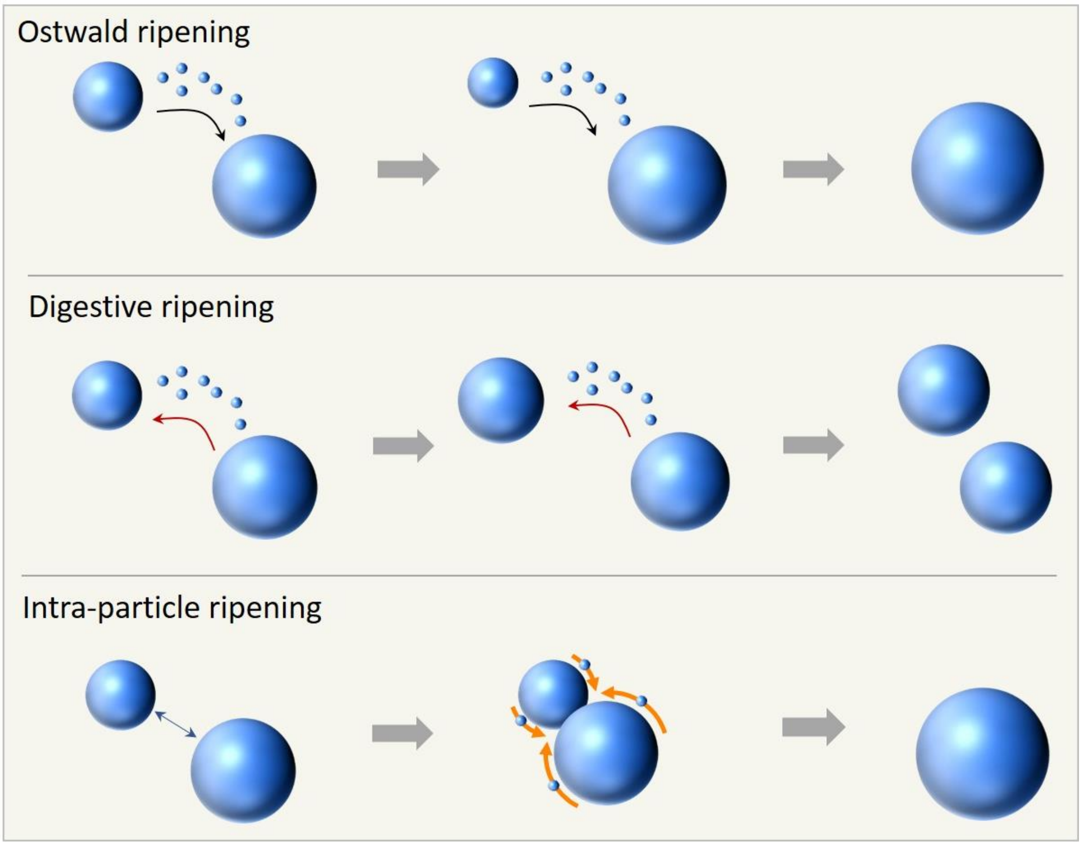

Supplementary Fig. 6: Schematic diagrams of three different ripening processes. For Ostwald ripening, the growth of larger particles is accompanied by the consumption of smaller particles, which eventually become larger particles. For digestive ripening, the smaller particles in the solution dissolve and redeposit on the larger particles resulting in uniform particle size distribution. For intra-particle ripening, the matter of two particles diffuses to the neck along the surface and becomes one large particle.

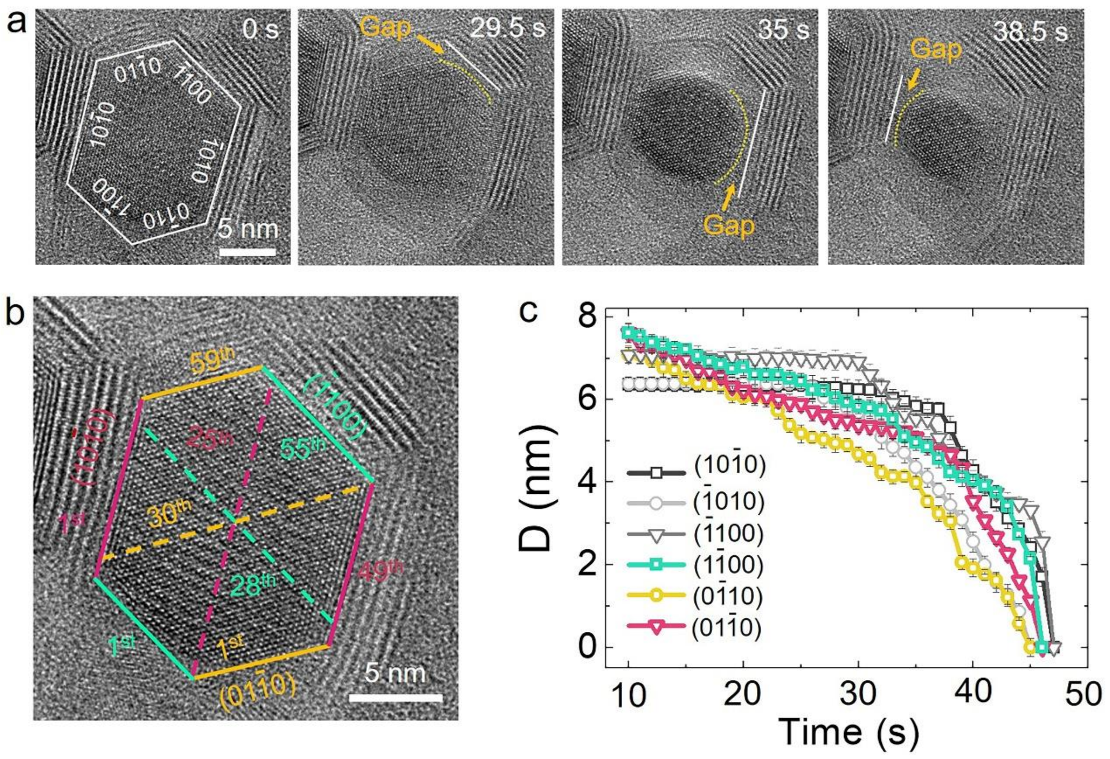

Supplementary Fig. 7. The dissolution of each crystal facet of particle 2. a, TEM images show the formation of gaps between core and shell for each side. At 0 seconds, each lattice facet is marked with a white line. From the images, we can detect when the gaps formed on each side. The gap is formed first at $(\bar{1}100)$ side, then at $(\bar{1}010)$, and finally at $(10\bar{1}0)$ side. **b,** Determination of geometric center for particle 2. We counted the number of atom layers for different facets (specifically $(10\bar{1}0)$, $(1\bar{1}00)$, and $(01\bar{1}0)$) and marked the middle layer by the dotted lines. The intersection of the three-dotted lines is treated as the center point of particle 2. **c,** The change of the vertical distance between the crystal center and each crystal facet with time. We can see the three facets with incomplete shells ($(1\bar{1}00)$, $(0\bar{1}10)$, and $(01\bar{1}0)$) dissolve much faster than the other three facets with the undamaged shell ($(10\bar{1}0)$, $(\bar{1}010)$, and $(\bar{1}100)$) before the gap formation. After the formation of gaps, the priority of dissolution disappears, all facets are dissolved quickly.

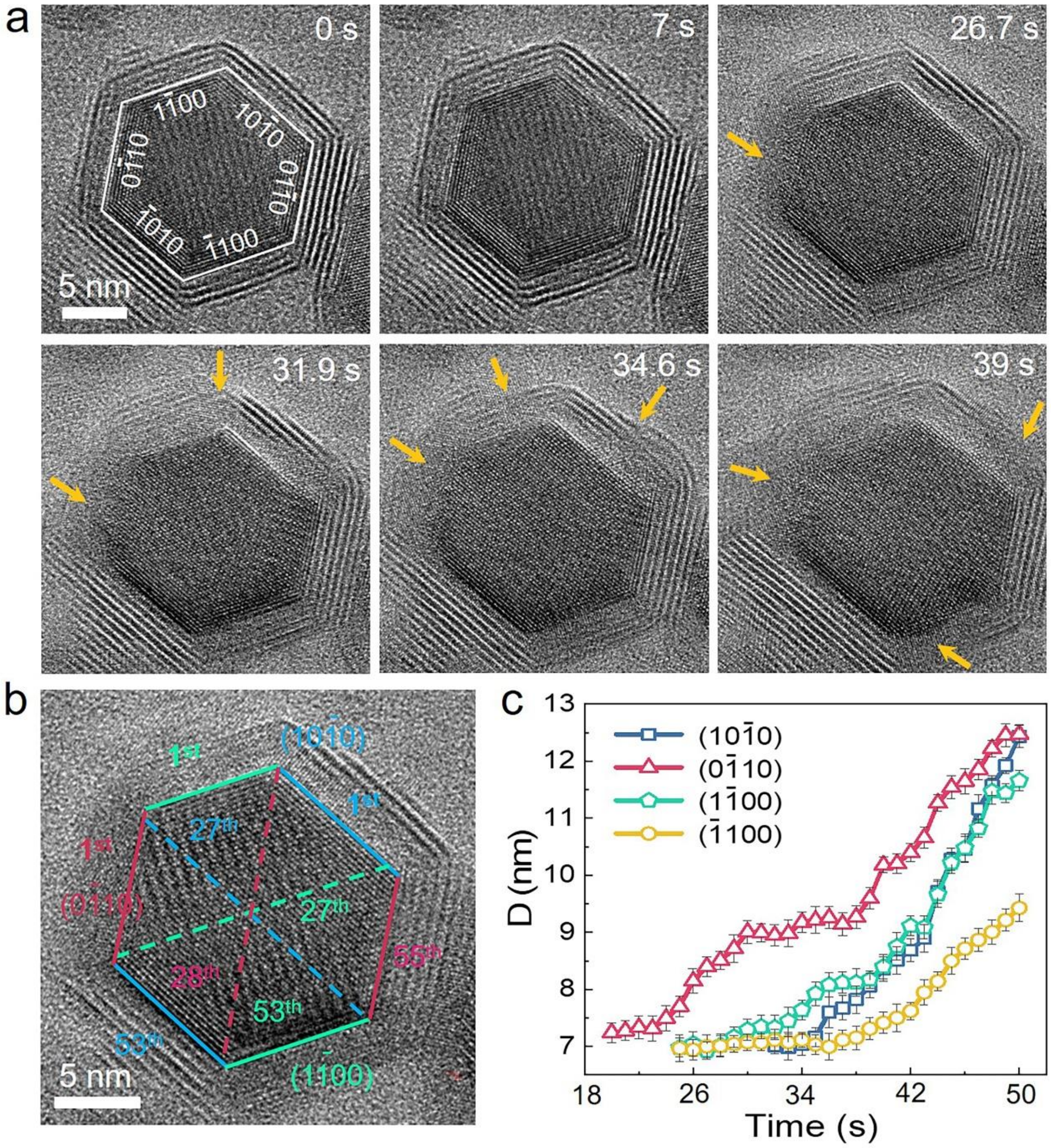

Supplementary Fig. 8. The growth of each crystal facet of particle 1. a, TEM images show the cracks in the shell for each side. At 0 seconds, each lattice facet is marked with a white line. The other images show when the cracks formed on each side. The cracks formed first at (0$\bar{1}$10) side, then at (1$\bar{1}$00), then at (10$\bar{1}$0), and finally at ($\bar{1}$100) side. **b,** Determination of geometric center for particle 1. We counted the number of atom layers for different facets (specifically (10$\bar{1}$0), (1$\bar{1}$00), and (0$\bar{1}$10)) and marked the middle layer by the dotted lines. The intersection of the three-dotted lines is treated as the center point of particle 1. **c,** The change of the vertical distance between the crystal center and each crystal facet with time. We can see the facets start to grow after cracks. The (0$\bar{1}$10) facet starts to grow first, followed by the (1$\bar{1}$00) and ($\bar{1}$100) facets, and finally the (10$\bar{1}$0) facet. The ($\bar{1}$010) and (01$\bar{1}$0) facets, with intact shells, do not grow.

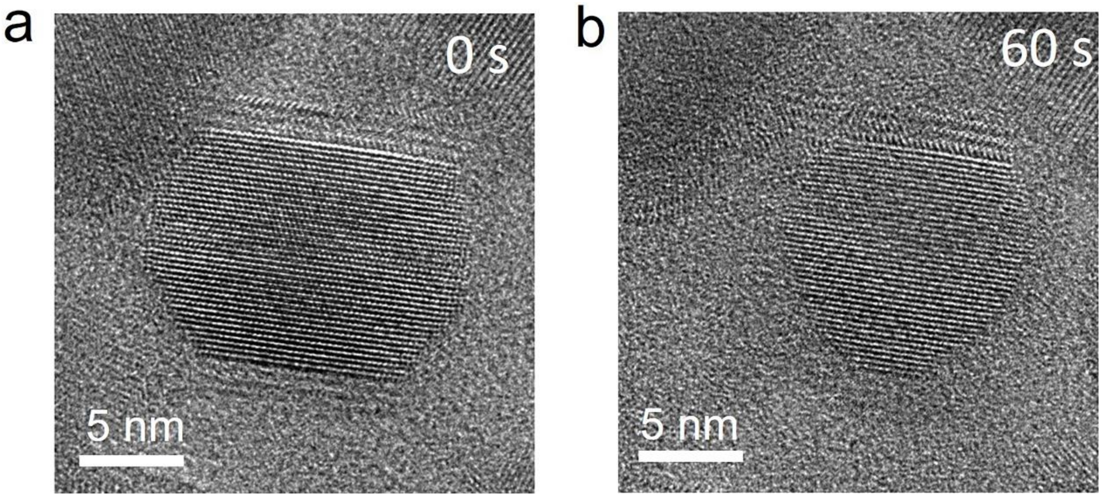

Supplementary Fig. 9. Dissolution of Cd-$CdCl_2$ core-shell particles can be in the solution spontaneously without electron beam irradiation. To exclude the influence of the electron beam on particle dissolution, we block the electron beam after we took the first image, then took another one after blocking for 60 s. **a,** TEM image of a particle before blocking electron beam. **b,** TEM image of the same particle after shutting off electron beam for 60 s. Comparing the shape of the sample particle in TEM images, we can see that the particle is still partially dissolved after 60 s even without electron beam irradiation.

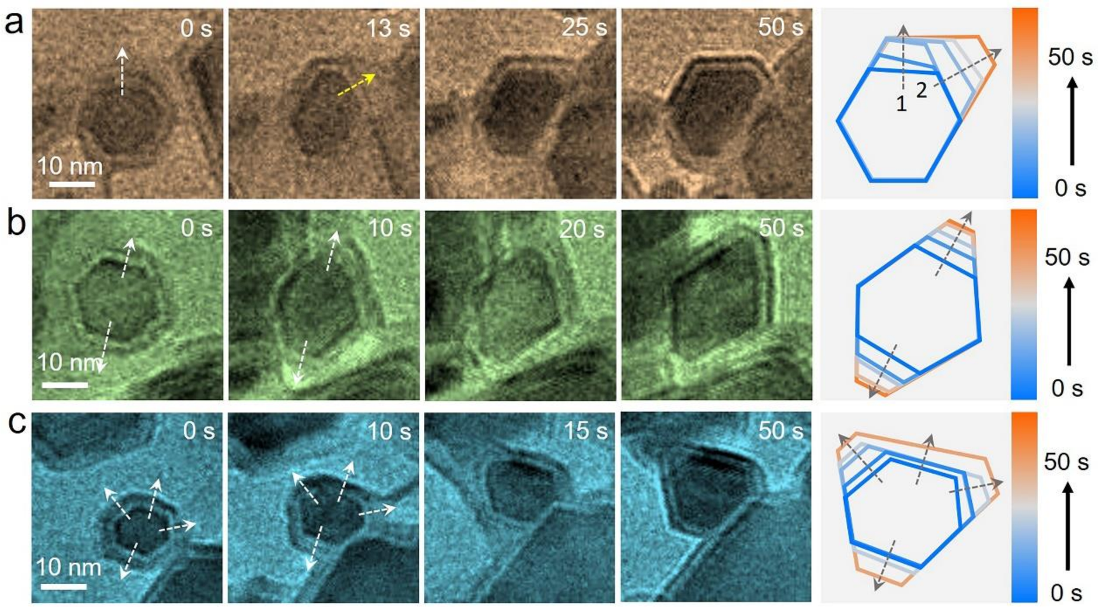

Supplementary Fig. 10: The sequence of in-situ TEM images showing the directional growth of regular hexagonal-shaped Cd-$CdCl_2$ core-shell structured particles. The rightmost column lists their corresponding shape contour evolution. **a,** Images show a regular hexagonal-shaped Cd-$CdCl_2$ core-shell particle first grows along one crystal facet as pointed by a white arrow and then along the other facet as point by a yellow arrow. Scale bar, 10 nm. **b,** Images show a regular hexagonal-shaped Cd-$CdCl_2$ core-shell particle grows along with two opposite directions as pointed by white arrows, forming a rhomboid-like shape. Scale bar, 10 nm. **c,** A regular hexagonal-shaped Cd-$CdCl_2$ core-shell particle grows directionally on the four crystal facets. Through directional growth of crystal facets, the shape of particles can convert among various hexagons. Scale bar, 10 nm.

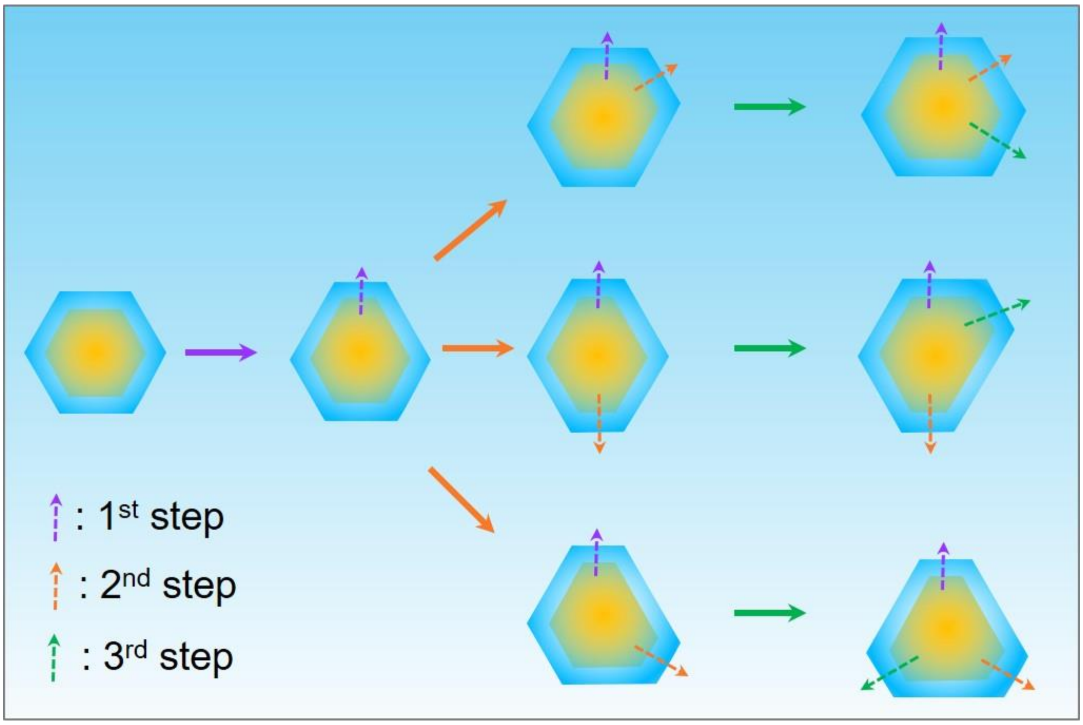

Supplementary Fig. 11: Promising strategies for shape-controlled growth of core-shell structured nanoparticles. During growth, the final shape of the particles can be predicted. By generating and repairing crack defects on selected facets, we can realize the growth of a single facet, two facets, and multiple facets of regular hexagonal core-shell particles.

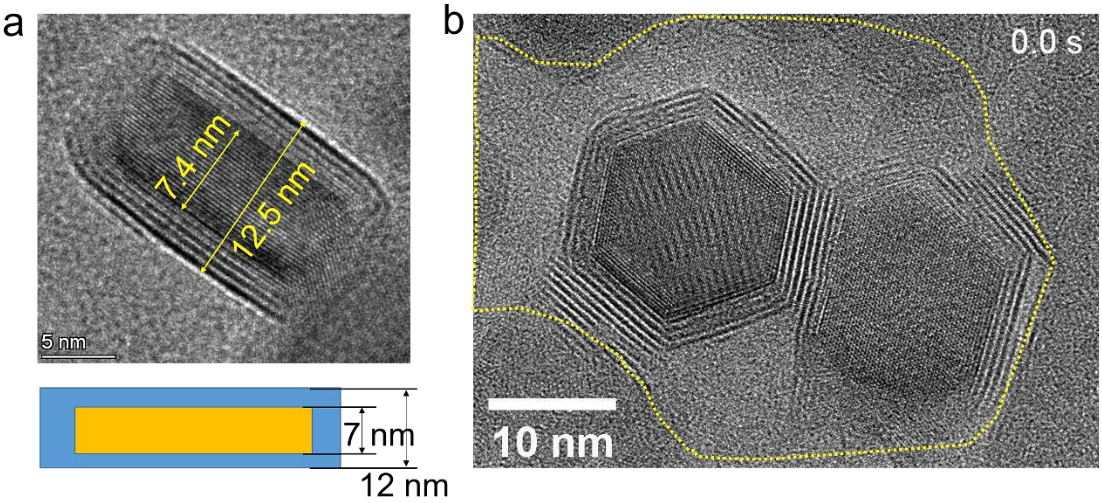

Supplementary Fig. 12: Estimation of core-shell particle parameters and solution volume. a, Measurement and estimation of core and shell thickness of nanoparticles. We first measured the thickness of a nanoplate and its core. Then we assume that the two particles have the same thickness. The thickness of the core is set to 7 nm, and the thickness of the nanoplate is set to 12 nm. **b,** Estimation of solution volume. The yellow line outlines the edge with liquid, which is concentrated in the area with weak contrast. The measured value of the yellow area is measured at about 650 nm^2. During the calculation of ions concentration, we treat the volume of the liquid as 13000 nm^3 and 50000 nm^3. Although the volume of the solution affects the absolute concentration of ions in the solution, it does not affect the changing trend of ions concentration in the solution. Therefore, we can qualitatively evaluate the changing trend of ion concentration in the solution.

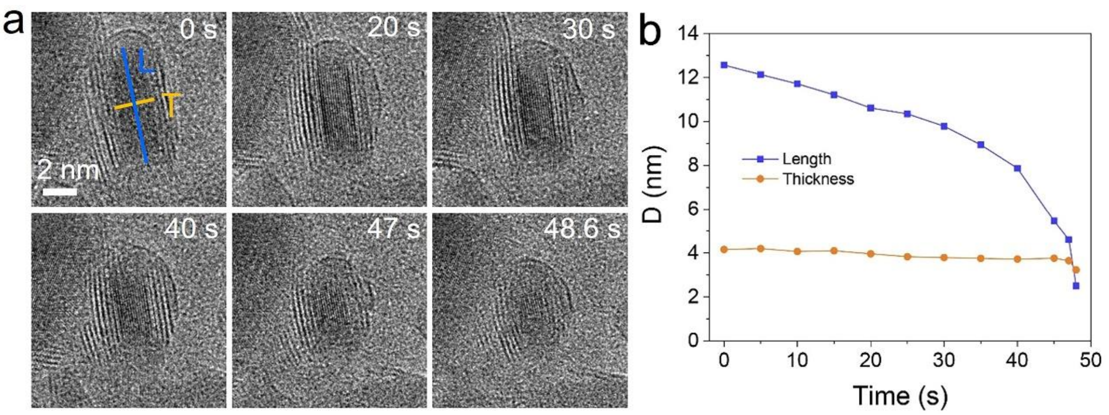

Supplementary Fig. 13: The shape evolution of a particle during ripening is viewed from the side. a, Sequential images show the shape evolution of one Cd-$CdCl_2$ core-shell particle from the side view. **b,** Changes in measured length and thickness. From the plot, we can see that two seconds before the complete disappearance, the dissolution mainly proceeds along the side surface, and the base surface hardly dissolves.

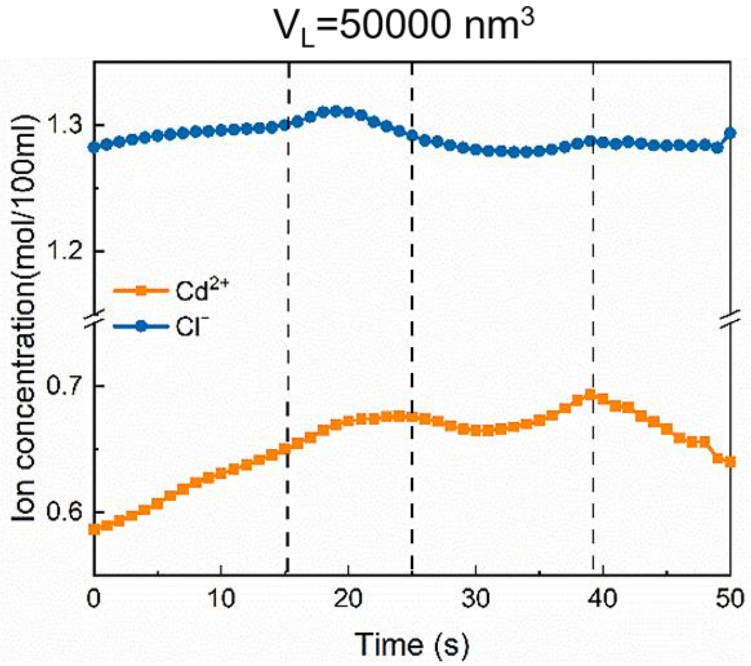

Supplementary Fig. 14: Ion concentration changes in the solution with time. The estimated liquid volume is 50000 nm^3. Initially, the ion concentration increases resulting in a solution change from unsaturated to saturated (0-16 s). Then, the ion concentration continued to increase, causing the $CdCl_2$ to rupture (16-25 s). After the crack defect formation, mass transfer from particle 1 to particle 2 (25-40 s). At the end of mass transfer, the ions in the supersaturated solution grow on particle 1, reducing the ion concentration (40-50 s).

Supplementary Table 1: Crystal structure data for Cd and $CdCl_2$. The entries in blue correspond to the materials in this report[1-3].

Chemical Formula	Structure	a (Å)	b (Å)	c (Å)	α (°C)	β (°C)	γ (°C)
**$CdCl_2$**	Trigonal	6.23	6.23	6.23	36.03	36.03	36.03
**$CdCl_2$**	Trigonal	3.8459	3.8459	17.4931	90	90	120
**Cd**	hexagonal	2.977	2.977	5.612	90	90	120

Supplementary Table 2: Parameters used for ion concentration estimation[4].

	Density (g/cm^3)	Molar mass (g/mol)	Solubility (mol/L)
**$CdCl_2$**	4.047	183.32	6.5×10^{-2}
**Cd**	8.65	112	insoluble

Supplementary References

1. Partin, DE, O'Keeffe, M. The structures and crystal chemistry of magnesium chloride and cadmium chloride. *J Solid State Chem* **95**, 176-183 (1991).
2. Yu, W., Zhao, M. Determination of the temperature variation of the crystal structure of $CdCl_2$ form the EPR data for Mn^{2+}. *J. phys., C, Solid state phys.* **19**, 6761-6766 (1986).
3. Rossmanith, E. X-ray measurement of the root-mean-square displacement of the atoms in cadmium single crystals. *Acta Cryst.*. **34**, 497-500 (1978).
4. Dean, J. Lange's handbook of chemistry McGrawhill book Co. Inc. (1999).
