# Supplementary material for: Defect-mediated ripening of core-shell nanostructures
Source: Nat Commun. 2022 Apr 25;13:2211. doi: 10.1038/s41467-022-29847-8 (PMC9038757; doi:10.1038/s41467-022-29847-8)
Supplement: Supplementary file 3 — Description of Additional Supplementary Files [file 41467_2022_29847_MOESM3_ESM.pdf]

Description of Additional Supplementary Files

Supplementary Movie 1: The defect-mediated ripening process of Cd-CdCl2 core-shell particles. The play rate is 50 frames/s.

Supplementary Movie 2: The dissolution of Cd core without the protection of CdCl2 shell. The play rate is 50 frames/s.

Supplementary Movie 3: The dissolution of Cd core with the protection of CdCl2 shell. The play rate is 50 frames/s.

Supplementary Movie 4: The structural evolution of shell before the formation of crack defects and the directional growth of the core. The play rate is 50 frames/s.

Supplementary Movie 5: The formation of Cd-CdCl2 core-shell nanostructured particles in the growth solution. The play rate is 50 frames/s.

Supplementary Movie 6: The sates of Cd-CdCl2 core-shell before ripening.
